# Late recurrence and histological progression of a cervical intradural extramedullary solitary fibrous tumor after gross total resection: a case report

**DOI:** 10.3389/fonc.2026.1859643

**Published:** 2026-06-15

**Authors:** Yergen N. Kenzhegulov, Daniyar K. Zhamoldin, Viktor G. Aleinikov, Talgat T. Kerimbayev, Berik Zhetpisbaev, Makar P. Solodovnikov, Aisa Z. Nurpeisov, Serik Akshulakov

**Affiliations:** 1 Department of Minimally Invasive Neurosurgery, Joint Stock Company (JSC) “National Centre for Neurosurgery”, Astana, Kazakhstan; 2 Department of Spinal Neurosurgery and Peripheral Nervous System Pathology, Joint Stock Company (JSC) “National Centre for Neurosurgery”, Astana, Kazakhstan; 3 Department of Pathology, Joint Stock Company (JSC) “National Centre for Neurosurgery”, Astana, Kazakhstan; 4 Department of Research Management, Joint Stock Company (JSC) “National Centre for Neurosurgery”, Astana, Kazakhstan; 5 Department of Anesthesiology, Resuscitation and Intensive Care, Joint Stock Company (JSC) “National Centre for Neurosurgery”, Astana, Kazakhstan; 6 Department of Brain Neurosurgery, Joint Stock Company (JSC) “National Centre for Neurosurgery”, Astana, Kazakhstan

**Keywords:** gross total resection, histological progression, intradural extramedullary tumor, solitary fibrous tumor, spinal tumor, STAT6, tumor recurrence

## Abstract

**Background:**

Solitary fibrous tumor/hemangiopericytoma (SFT) is a rare mesenchymal neoplasm of the central nervous system characterized by unpredictable biological behavior, including delayed recurrence and metastatic potential. Spinal localization is uncommon, while cervical intradural extramedullary involvement remains exceptionally rare. Long-term outcomes and optimal postoperative management strategies are not well established.

**Case presentation:**

A 39-year-old male presented with progressive cervical myelopathy caused by an intradural extramedullary tumor at the C4–C5 level. The patient underwent microsurgical gross total resection (GTR) with intraoperative neurophysiological monitoring. Histopathological and immunohistochemical evaluation demonstrated a CNS WHO grade 2 solitary fibrous tumor with diffuse CD34 and STAT6 positivity. Postoperative recovery was uneventful, and serial contrast-enhanced magnetic resonance imaging (MRI) demonstrated no evidence of recurrence during a 5-year surveillance period.

**Results:**

In 2026, the patient developed recurrent cervical pain and sensory disturbances. Follow-up MRI revealed a localized recurrent intradural extramedullary lesion at the previous surgical site. Systemic staging showed no evidence of metastatic disease. Repeat microsurgical gross total resection was performed. Histopathological examination of the recurrent lesion demonstrated increased mitotic activity (>5 mitoses per 10 high-power fields), tumor necrosis, and an elevated Ki-67 proliferative index (10–12%), consistent with progression to CNS WHO grade 3 solitary fibrous tumor. Postoperative MRI confirmed complete tumor removal. Following multidisciplinary evaluation, close radiological surveillance was recommended.

**Conclusion:**

This case highlights the potential for delayed recurrence and histological progression of spinal solitary fibrous tumors despite apparently curative gross total resection and prolonged disease-free survival. Long-term, potentially lifelong radiological follow-up should be considered even for initially low-grade lesions. Recurrent or higher-grade tumors may require multidisciplinary evaluation regarding adjuvant treatment strategies and systemic surveillance.

## 1 Introduction

Solitary fibrous tumor (SFT), a unified entity encompassing tumors previously classified as hemangiopericytoma in earlier WHO classifications of central nervous system tumors, is a rare fibroblastic mesenchymal neoplasm characterized by NAB2–STAT6 fusion, nuclear STAT6 expression, branching “staghorn” vasculature, and variable biological behavior (1–3). Spinal involvement is uncommon, accounting for only a small proportion of CNS SFT cases, while cervical intradural extramedullary localization remains exceptionally rare (4, 5).

Although gross total resection (GTR) is considered the mainstay of treatment, these tumors demonstrate unpredictable biological behavior with potential for late local recurrence and distant metastasis, even years after apparently complete removal (4, 6). Histological progression toward higher WHO grades during recurrence has also been reported and may indicate more aggressive clinical behavior (6, 7).

Because of the rarity of spinal SFT, long-term outcomes and optimal postoperative management remain insufficiently defined, particularly for cervical lesions. We report a rare case of delayed recurrence of a cervical intradural extramedullary SFT occurring five years after GTR, accompanied by histological progression from WHO grade 2 to grade 3. The case highlights the importance of prolonged surveillance and illustrates the potential for biological transformation despite an initially favorable postoperative course.

## 2 Case presentation

A 39-year-old male presented with progressive neurological symptoms. His past medical history was significant for arterial hypertension grade I (risk 2), chronic hepatitis C (minimal activity), and chronic erosive gastritis in partial remission. In 2004, he underwent exploratory laparotomy following a motor vehicle accident with prior blood transfusion. Family and allergic histories were unremarkable.

The duration of symptoms was approximately 7 months. Initial manifestations began in April 2021 with neck pain and upper extremity sensory disturbances, followed by gradual neurological deterioration.

### 2.1 Clinical presentation

At admission in November 2021, the patient presented with persistent cervical pain and progressively worsening motor weakness in the upper extremities, accompanied by increasing weakness in the left lower limb. The patient also reported sensory disturbances in both upper extremities, generalized fatigue, and headache.

Because the mJOA score was not documented in the original records, retrospective functional assessment was based on ambulatory status and detailed neurological examination findings. On neurological examination, the patient was fully conscious with a Glasgow Coma Scale score of 15. A marked spasm of the cervical paraspinal muscles was noted. Motor assessment demonstrated reduced strength in the upper extremities graded at 4–4.5/5 on the Medical Research Council (MRC) scale. Sensory examination revealed decreased superficial sensation in both upper extremities and in the left lower limb, as well as impaired deep sensation in the left-sided extremities. Deep tendon reflexes were brisk, with expanded reflexogenic zones, more pronounced on the right side than the left (D > S). No sphincter dysfunction was observed.

Overall, the clinical findings were consistent with progressive cervical myelopathy. The modified Japanese Orthopedic Association (mJOA) score was not documented in the medical records.

MRI of the cervical spine (15.11.2021) demonstrated a well-defined intradural extramedullary mass at the C4–C5 level causing significant spinal cord compression. The lesion appeared isointense on T1-weighted images and iso- to slightly hyperintense on T2-weighted sequences, with homogeneous contrast enhancement following gadolinium administration. No clear dural tail sign was identified. Mild intramedullary T2 hyperintensity suggested compressive myelopathy.

### 2.2 Surgical procedure

#### 2.2.1 Procedure

Posterior midline approach C4–C5; left-sided hemilaminectomy C4–C5; longitudinal durotomy; microsurgical gross total resection of intradural extramedullary tumor at C3–C5 with intraoperative neurophysiological monitoring.

Surgery was performed under general combined anesthesia with the patient in the prone position. Prophylactic intravenous cefazolin (2.0 g) was administered prior to incision. Intraoperative neurophysiological monitoring (IONM) electrodes were placed for continuous assessment of spinal cord function. Under fluoroscopic guidance, the C4–C5 level was identified and marked. After standard sterile preparation with povidone-iodine solution, a 4-cm midline skin incision was made over the C4–C5 spinous processes. The paraspinal muscles were dissected subperiosteally, and a self-retaining retractor (Jansen) was applied. An operating microscope was introduced. A left-sided hemilaminectomy of C4 and C5 was performed using a high-speed drill and 3.0-mm Kerrison rongeurs. The dura mater appeared tense with decreased pulsation. A linear longitudinal durotomy was carried out. Intradural exploration under microscopic magnification and continuous IONM revealed a well-vascularized, dirty bluish-colored extramedullary mass extending inferiorly below the C3 level and oriented in a lateromedial direction. The tumor was adherent to the spinal cord surface and dura mater but clearly distinguishable from nerve roots and surrounding neural tissue. Using meticulous microsurgical dissection and bipolar coagulation, the lesion was progressively devascularized and separated from adjacent structures. A distinct dissection plane was identified, allowing for safe circumferential mobilization. The tumor was removed en bloc within the limits of visual and anatomical accessibility. Throughout resection, no pathological signal changes were detected on intraoperative neurophysiological monitoring. Hemostasis was achieved using bipolar coagulation, fibrillar hemostatic agents, and TachoComb plates. The operative field was dry. The dura mater was closed in a watertight fashion. Layered wound closure was performed. Estimated blood loss was approximately 50 mL. Resected tissue was sent for histopathological examination.

#### 2.2.2 Extent of resection

Gross total resection (GTR), later confirmed by postoperative contrast-enhanced MRI. The early postoperative course was uneventful. The patient was mobilized on postoperative day 2.

Contrast-enhanced MRI of the cervical spine performed on postoperative day 1 demonstrated radiological changes consistent with recent surgical intervention at the C4–C5 level. A postoperative cavity was identified at the site of tumor resection, filled with cerebrospinal fluid (CSF)-intensity content on all sequences, without evidence of residual mass lesion. No nodular or focal contrast-enhancing component suggestive of residual tumor was observed. The spinal cord was displaced anteriorly and closely apposed to the anterior wall of the spinal canal. The cord contour appeared irregular and indistinct. Intramedullary hyperintense foci on T2-weighted imaging were noted, most likely reflecting postoperative edema and/or chronic demyelinating changes secondary to long-standing compression. In the posterior cervical and upper thoracic regions, as well as at the sites of resected posterior elements, postoperative soft tissue thickening was observed without radiological signs of hemorrhagic collection. Following contrast administration, linear enhancement was detected along the dura mater, consistent with expected postoperative dural enhancement. No abnormal intramedullary or extramedullary pathological enhancement was identified.

#### 2.2.3 Radiological conclusion

Findings consistent with gross total resection of intradural extramedullary tumor at C4–C5. Postoperative changes without evidence of residual tumor or acute hemorrhagic complications.

### 2.3 Histopathological findings

Histopathological examination of the resected specimen revealed a moderately cellular spindle-cell neoplasm composed of uniform oval-to-spindle-shaped cells arranged in short fascicles and patternless architecture within a collagenous stroma. The tumor demonstrated a prominent branching vascular network with thin-walled, ectatic vessels consistent with a so-called “staghorn” hemangiopericytoma-like vascular pattern. No overt tumor necrosis was identified. Mitotic activity was consistent with an intermediate-grade lesion (corresponding to WHO grade II), with a mitotic index of less than 5%. Immunohistochemical analysis showed: Strong nuclear expression of STAT6; Diffuse positivity for CD34. This immunophenotypic profile supports the diagnosis of SFT within the current WHO classification framework. Final diagnosis: Intradural extramedullary SFT(CD34+, STAT6+), WHO Grade II, ICD-O code 8815/1.

(Original preoperative imaging from 2021 was not available for direct review; however, detailed radiological reports and early postoperative contrast-enhanced MRI confirming gross total resection were available).

### 2.4 Postoperative course (2021–2026)

The early postoperative period was uneventful. The patient demonstrated gradual neurological improvement, with partial regression of sensory deficits and stabilization of motor function. At discharge, he was ambulatory without assistance, and the surgical wound healed by primary intention. Following histopathological confirmation of a WHO grade II SFT and documentation of gross total resection (GTR) on early postoperative contrast-enhanced MRI, the patient was referred to a medical oncologist for further evaluation. However, based on available medical documentation and considering the completeness of resection, adjuvant radiotherapy or other forms of specific oncological treatment were not recommended.

The patient remained under regular follow-up with a neurosurgeon. Serial contrast-enhanced MRI examinations were performed according to a surveillance protocol (at 3 months, 12 months, and subsequently annually). Follow-up imaging demonstrated no evidence of local recurrence or residual tumor growth. Throughout the observation period, the patient underwent structured neurorehabilitation, including physiotherapy and therapeutic exercise programs. Neurological status remained stable with sustained functional recovery. No new neurological deficits or sphincter disturbances were reported. As of the latest follow-up in 2026, the patient remains clinically stable with no radiological signs of recurrence.

Disease-free interval: 5 years following gross total resection.

## 3 Recurrence

At follow-up, the patient presented with new neurological complaints consisting of persistent cervical and upper thoracic pain, accompanied by intermittent numbness in the left upper extremity.

A contrast-enhanced MRI of the cervical spine performed on 21.01.2026 demonstrated radiological evidence of tumor recurrence at the C4–C5 level (**Figure 1**). MRI revealed a well-defined, solid, rounded lesion measuring 11.0 × 12.3 × 8.3 mm, located along the posterior surface of the dura mater at the C4–C5 vertebral level. The mass demonstrated broad-based attachment to the dorsal dura and close adherence to the spinal cord and adjacent nerve roots. The lesion appeared hyperintense on T2-weighted images and exhibited marked, homogeneous contrast enhancement following gadolinium administration, consistent with a hypervascular solid tumor. Radiological characteristics were highly suggestive of local recurrence of SFT. No distant spinal lesions were identified at that time.

**Figure 1:**
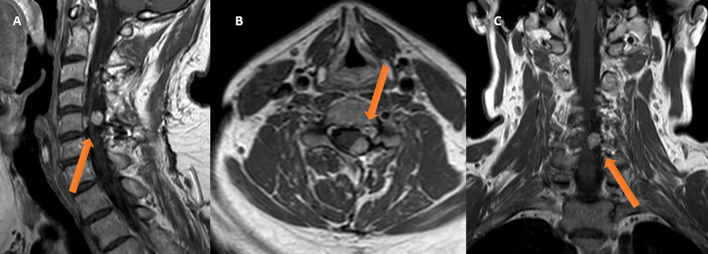
Contrast-enhanced MRI of the cervical spine demonstrating tumor recurrence at C4–C5(*orange arrow*). Post-contrast T1-weighted MRI (sagittal **(А)**, axial **(В)**, and coronal **(С)** views) shows a well-defined intradural extramedullary lesion along the dorsal dura at the C4–C5 level measuring 11.0 × 12.3 × 8.3 mm with intense homogeneous enhancement, causing mild anterior displacement of the spinal cord and consistent with local recurrence of SFT.

Radiological conclusion: Local intradural extramedullary recurrence at C4–C5 with dural attachment and involvement of adjacent neural structures. At the time of recurrence, systemic staging was performed using contrast-enhanced CT of the chest and abdomen, which revealed no evidence of extracranial metastatic disease. The case was discussed at a multidisciplinary tumor board involving neurosurgery, neuroradiology, pathology, and oncology specialists. Considering the localized recurrence and feasibility of complete microsurgical excision, repeat gross total resection was recommended.

### 3.1 Second surgery

The procedure was performed under general endotracheal anesthesia with the patient in the prone position. Prophylactic intravenous cefazolin (2000 mg) was administered, and continuous intraoperative neurophysiological monitoring (IONM) was used. A 6-cm midline incision was made along the previous scar over the C4–C6 levels. Dense subcutaneous, intermuscular, and epidural scar tissue with keloid formation was encountered, requiring meticulous microsurgical dissection. After skeletonization of C4–C6 posterior elements, the dura mater was exposed. It appeared tense with weak pulsation, and hypertrophied dural vessels caused significant bleeding, controlled with bipolar coagulation. Following longitudinal durotomy (C4–C6), clear cerebrospinal fluid was released. An intradural extramedullary mass was identified. The tumor was burgundy-red, soft-elastic, encapsulated, and markedly hypervascular, with dense adhesions to the spinal cord, dura, and nerve roots. Prominent abnormal vascular structures were observed. (**Figure 2**).

**Figure 2:**
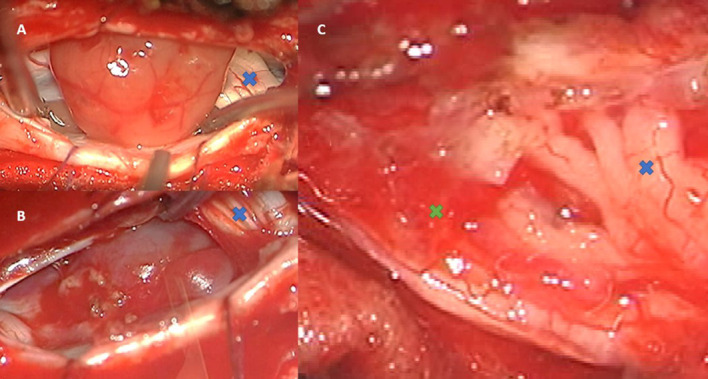
Intraoperative appearance during repeat microsurgical resection. **(A)** After longitudinal durotomy, a well-vascularized, reddish nodular tumor is visualized compressing the spinal cord within the intradural space. **(B)** A separate tumor nodule (“satellite” component) identified beneath the nerve roots(*blue marker*), demonstrating a lobulated configuration and close subradicular location. **(C)** Advanced stage of microsurgical dissection showing the tumor densely adherent and infiltrating between multiple nerve roots, with radially oriented pathological vessels extending from the central tumor mass toward the periphery, creating an “octopus-like” vascular configuration(*green marker*).

Microsurgical resection was performed using bipolar coagulation and aspiration under continuous IONM guidance. Despite pronounced vascularity and adhesions, careful identification of neural structures allowed safe circumferential dissection. No intraoperative neurophysiological deterioration was detected. The tumor was removed completely within the limits of anatomical accessibility. Hemostasis was achieved using bipolar coagulation and hemostatic agents. The dura was closed watertight, and the wound was sutured in layers.

Extent of resection: Gross total resection (GTR). **Figure 3** (Post-operative contrast enhanced MRI).

**Figure 3:**
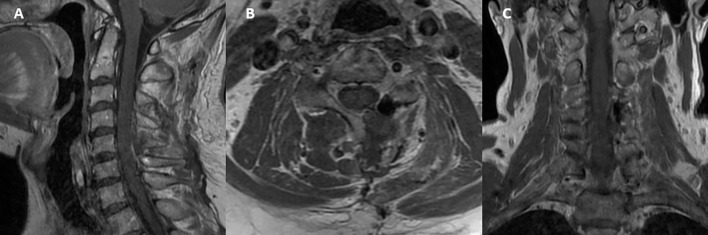
Early postoperative contrast-enhanced MRI of the cervical spine (postoperative day 1). T1-weighted contrast-enhanced MRI (sagittal **(А)**, axial **(В)**, and coronal **(С)** views) demonstrates postoperative changes after C4–C5 laminectomy and removal of an intradural extramedullary tumor, including soft tissue thickening within the surgical bed without signs of hemorrhage. The subcutaneous fat layer is thickened and edematous with small foci of postoperative air. Peripheral contrast enhancement is observed along the margins of the surgical cavity and along the operative corridor.

### 3.2 Histology of recurrence

#### 3.2.1 Macroscopic findings

The submitted specimen consisted of multiple small fragments of gray soft tissue measuring up to 0.2 × 0.2 × 0.1 cm, with a soft consistency. (**Figure 4**).

**Figure 4:**
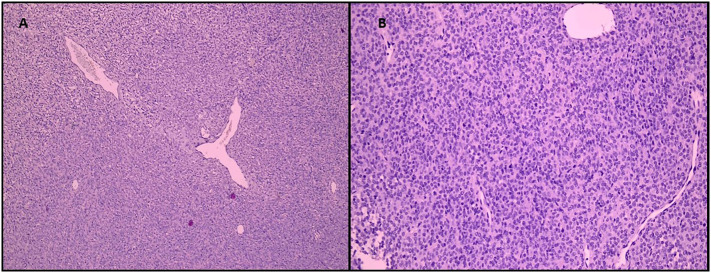
Histopathological features of the solitary fibrous tumor. **(A)** Low-power photomicrograph showing a spindle-cell neoplasm composed of relatively uniform cells arranged in short fascicles and a patternless architecture within a collagenous stroma, with characteristic branching hemangiopericytoma-like vessels. Hematoxylin and eosin (H&E) stain, ×100. **(B)** High-power view demonstrating oval-to-spindle tumor cells with mildly hyperchromatic nuclei and scant cytoplasm surrounding the characteristic branching vascular structures. Hematoxylin and eosin (H&E) stain, ×200.

#### 3.2.2 Microscopic findings

Histological examination of hematoxylin–eosin stained sections demonstrated fragments of tumor tissue composed of relatively dense proliferations of round, oval, and focally spindle-shaped cells with hyperchromatic nuclei. Numerous mitotic figures (>5 mitoses per 10 high-power fields) were identified. Tumor cells showed eosinophilic cytoplasm with indistinct cell borders. The tumor exhibited a prominent vascular pattern, including numerous sinusoidal vascular spaces lined by endothelium, as well as characteristic branching vessels with “staghorn” morphology. In addition, extensive areas of coagulative necrosis and focal hemorrhages were observed. (**Figure 4**).

Immunohistochemistry: Immunohistochemical analysis demonstrated: CD34: predominantly diffuse positive expression; STAT6: weak but diffuse nuclear positivity; Progesterone receptor (PR): negative; Ki-67: heterogeneous proliferative index with focal areas reaching 10–12%. (**Figure 5**).

**Figure 5:**
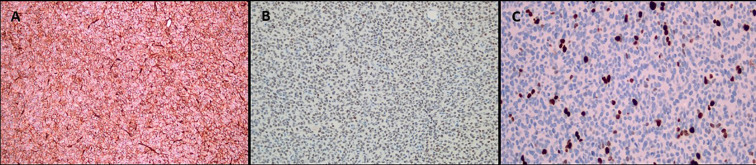
Immunohistochemical features of solitary fibrous tumor. **(A)** Tumor cells demonstrating diffuse positive immunoreactivity for CD34. Immunohistochemical staining, ×200. **(B)** Diffuse nuclear positivity for STAT6, supporting the diagnosis of solitary fibrous tumor. Immunohistochemical staining, ×200. **(C)** Ki-67 proliferative index approximately 10–12%, indicating increased proliferative activity of tumor cells. Immunohistochemical staining, ×200.

Taken together, the recurrent lesion demonstrated histomorphological features similar to the primary tumor, including spindle-cell proliferation and the characteristic hemangiopericytoma-like vascular pattern. However, the recurrent tumor showed increased proliferative activity and the presence of tumor necrosis, findings that were not reported in the primary specimen. According to the current WHO classification, the presence of necrosis and increased mitotic activity supports classification as a higher-grade tumor. In the present case, the recurrent tumor demonstrated >5 mitoses per 10 HPF and extensive necrosis, with an increased Ki-67 index (up to 10–12%), supporting classification as CNS WHO grade 3 solitary fibrous tumor.

Final diagnosis; Recurrent solitary fibrous tumor (previously hemangiopericytoma), CNS WHO grade 3, ICD-O code 8815/1, showing increased mitotic activity and necrosis compared with the primary lesion.

Following histopathological confirmation of CNS WHO grade 3 recurrent SFT, the patient was referred for oncological evaluation and multidisciplinary discussion regarding adjuvant radiotherapy. Although adjuvant radiotherapy was considered because of the higher tumor grade and recurrent nature of the lesion, it was not initiated at that stage due to achievement of gross total resection, absence of radiological evidence of residual or metastatic disease, and the lack of definitive evidence regarding the benefit of postoperative radiotherapy after complete excision of spinal SFT. Close radiological surveillance was therefore recommended.

## 4 Discussion

SFT(SFT) is a rare mesenchymal neoplasm of fibroblastic origin that may arise in multiple anatomical locations, including the pleura, soft tissues, and the central nervous system (CNS). Within the CNS, these tumors account for less than 1% of primary intracranial neoplasms and an even smaller proportion of spinal tumors. Consequently, current understanding of their biological behavior and optimal management remains largely based on retrospective studies and isolated case reports.

In the spinal compartment, SFT most commonly arises from the spinal meninges and usually presents as an intradural extramedullary lesion, whereas purely intramedullary tumors are exceptionally rare. Clinical manifestations are generally related to progressive spinal cord compression and include axial pain, radiculopathy, and myelopathy. In a multicenter cohort study, spinal pain was reported in 69% of patients, while 41% presented with symptoms of spinal cord compression (4). Compared with intracranial counterparts, spinal SFT appears to demonstrate more favorable outcomes, with reported 10-year overall survival rates of approximately 85% versus 58% for tumors arising elsewhere in the CNS (3). Earlier clinical detection secondary to spinal cord compression and the greater likelihood of achieving gross total resection (GTR) may partially explain these differences. Nevertheless, the rarity of spinal SFT continues to hinder the development of standardized treatment algorithms and reliable prognostic models.

Histological behavior appears to significantly influence prognosis. Tumors coded as malignant (/3) demonstrate an approximately 40% higher mortality risk compared with benign (/0) lesions, although these categories do not directly correspond to WHO grades I–III (8). SFTs primarily metastasize hematogenously, most commonly to the lungs, whereas lymphatic spread is uncommon (9). According to the 2020 WHO classification, SFT is categorized as an intermediate fibroblastic neoplasm because of its potential for local recurrence and distant metastasis. Importantly, no metastatic disease was identified in the present case. However, following recurrence with progression to CNS WHO grade 3, closer long-term systemic surveillance may be justified given the increased metastatic potential associated with higher-grade tumors.

By 2021, approximately 50 cases of spinal SFT had been reported in the literature (Dincer et al., 2021) (10). More recent publications have further emphasized the heterogeneous and unpredictable behavior of these tumors, including delayed recurrence despite GTR, histological progression, and metastatic spread. Colamaria et al. reported variable clinical behavior depending on tumor grade and local invasion (Colamaria et al., 2022) (11), while Shidoh et al. described recurrence occurring 19 years after gross total resection, highlighting the necessity for lifelong radiological surveillance (Shidoh et al., 2024) (12). Other authors have also discussed the management challenges associated with recurrent or high-grade spinal SFT and the potential role of adjuvant radiotherapy in maintaining local tumor control (Tomomatsu et al., 2022; Alajmi et al., 2025) (13, 14). Collectively, these reports reinforce the persistent risk of recurrence and the unpredictable long-term course of spinal SFTs.

One of the principal diagnostic challenges is the absence of pathognomonic radiological features. On MRI, spinal SFTs are typically described as well-circumscribed intradural extramedullary masses with homogeneous or heterogeneous contrast enhancement, findings that substantially overlap with meningiomas and peripheral nerve sheath tumors (15, 16). In many cases, lesions appear isointense on both T1- and T2-weighted sequences with uniform post-contrast enhancement, further complicating differentiation from meningioma (15). Additional diagnostic complexity arises from the historical overlap with hemangiopericytoma, which is now unified with SFT following identification of the NAB2–STAT6 fusion (6, 17). Differential diagnosis may also include hemangioblastoma and metastatic lesions (18). Therefore, definitive diagnosis relies on histopathological and immunohistochemical evaluation. Histologically, SFTs are characterized by spindle-cell proliferation arranged in a patternless architecture with variable collagen deposition and characteristic “staghorn” branching vasculature (14, 19). Immunohistochemistry plays a central diagnostic role, with nuclear STAT6 expression representing a highly sensitive and specific marker associated with the NAB2–STAT6 fusion (19).

Surgical resection remains the cornerstone of treatment for spinal SFTs, particularly for intradural extramedullary lesions causing compression of the spinal cord and nerve roots. Surgery therefore serves both diagnostic and therapeutic purposes by providing histological confirmation and neural decompression. The primary surgical objective is safe GTR whenever feasible, as complete excision has consistently been associated with improved local control and lower recurrence rates (21, 22). Most reported cases are managed through laminectomy or hemilaminectomy, often resulting in significant postoperative neurological improvement (14, 23).

Nevertheless, surgical resection may be technically demanding because of tumor vascularity, firm consistency, and adhesions to the spinal cord or pia mater (17, 24). In some cases, the absence of a clear surgical plane necessitates meticulous microsurgical technique to minimize neurological morbidity. When GTR cannot be safely achieved, subtotal resection may be performed to preserve neurological function, although this approach is associated with a higher risk of recurrence (25). In such situations, adjuvant radiotherapy has been proposed to improve local control, particularly in residual or high-grade tumors (23, 26). Despite successful surgical treatment, delayed recurrence remains a recognized feature of SFT, with cases reported many years after initial resection (12). Accordingly, prolonged radiological surveillance is strongly recommended.

Spinal SFTs demonstrate an unpredictable clinical course with a substantial risk of delayed recurrence, even after apparently complete surgical removal. Several studies have reported recurrence occurring more than a decade after surgery, reflecting the slow-growing yet persistent biological behavior of these tumors (12, 27, 28). Histopathological grading appears to correlate with clinical aggressiveness, as higher-grade tumors are associated with increased recurrence, progression, and metastatic potential (7, 10). In the present case, the initial low-grade lesion remained stable for five years after GTR before recurring with progression to CNS WHO grade 3, supporting the association between histological progression and more aggressive biological behavior.

A multicenter analysis reported recurrence in approximately 43% of patients, with a mean recurrence interval of 5.8 years (4). Similar recurrence rates have been observed in other studies, particularly in cases involving subtotal resection or higher-grade histology (25). Extent of resection therefore remains one of the most important prognostic factors influencing long-term outcome, with subtotal resection significantly increasing the likelihood of tumor regrowth (21, 25). Although distant metastases are uncommon, they may occur in higher-grade tumors and most frequently involve the lungs, liver, or bones (23). Consequently, lifelong clinical and radiological follow-up is recommended, even after apparently complete tumor excision.

The role of radiotherapy in spinal SFT remains controversial because of the rarity of the disease and the limited availability of large clinical studies. Surgical resection remains the primary treatment modality, whereas radiotherapy is generally reserved for selected cases. Adjuvant radiotherapy is most commonly considered following subtotal resection, recurrent disease, or tumors demonstrating aggressive histological features (20, 21, 26). Several studies have suggested improved progression-free survival in recurrent or high-grade SFT treated with postoperative radiotherapy (16, 29), whereas its benefit following complete resection remains uncertain (20).

In the present case, recurrence occurred five years after initial GTR and was associated with progression to CNS WHO grade 3, including increased cellularity and necrosis, suggesting a more aggressive biological phenotype at recurrence. Although repeat surgery achieved complete radiological resection and the patient remained neurologically stable postoperatively, the potential role of adjuvant radiotherapy was considered because of the higher-grade recurrent disease. However, given the absence of residual tumor on postoperative MRI and the limited evidence supporting radiotherapy after repeat GTR in spinal SFTs, close radiological surveillance was ultimately selected.

Considering the recognized risk of delayed extracranial metastasis, baseline systemic staging should be considered, particularly in patients with recurrent disease, high-grade tumors, or aggressive histological features. Contrast-enhanced CT of the chest and abdomen may be sufficient in many cases, whereas PET/CT may be reserved for selected high-risk patients or when metastatic disease is clinically suspected. However, routine serial PET/CT surveillance after complete resection remains insufficiently supported by current evidence.

## 5 Limitations

This study has several limitations. First, it represents a single clinical case, which limits the generalizability of the findings and precludes definitive conclusions regarding optimal management strategies for spinal SFT(SFT). Given the rarity of intradural spinal SFT, current evidence remains largely limited to isolated case reports and small series. Second, although long-term follow-up was available, recurrence occurred after five years, while previous reports have demonstrated that SFT may recur decades after initial treatment. Therefore, even longer surveillance is necessary to fully assess long-term tumor behavior and treatment outcomes. Third, molecular genetic testing was not performed. Although STAT6 nuclear positivity strongly supported the diagnosis, confirmation of the NAB2–STAT6 fusion could have provided additional diagnostic and prognostic information. Finally, treatment decisions were made within a single institutional setting, and adjuvant radiotherapy was not administered after the initial gross total resection because of the absence of residual disease and the lack of established consensus guidelines. Consequently, the potential impact of adjuvant therapy on recurrence risk remains uncertain.

Despite these limitations, this report contributes to the limited literature on recurrent cervical spinal SFT by highlighting the potential for delayed recurrence and the importance of prolonged postoperative surveillance, even after apparently complete tumor resection.

## 6 Conclusion

SFT(SFT) of the cervical spine is a rare neoplasm with unpredictable biological behavior despite apparently successful gross total resection. The present case demonstrates that delayed local recurrence may occur even after a prolonged disease-free interval and may be accompanied by histological progression toward a higher WHO grade.

This report highlights the importance of long-term, potentially lifelong radiological surveillance in patients with spinal SFT, regardless of the extent of initial resection. Early detection of recurrence is critical for timely intervention and prevention of progressive neurological deterioration. In addition, recurrent or higher-grade tumors may warrant multidisciplinary evaluation and consideration of adjuvant treatment strategies, including radiotherapy, to optimize local disease control. Further accumulation of long-term clinical data is required to better define prognostic factors and postoperative management strategies for these rare tumors.

## Data Availability

The original contributions presented in the study are included in the article/supplementary material. Further inquiries can be directed to the corresponding author.
